# Dr. George Abbott

**Published:** 1911-05

**Authors:** 


### OBITUARY

Dr. George Abbott of Hamburg, N. Y., died at his home March 27, 1911, aged 84 years. Dr. Abbott had been a resident of Hamburg for 58 years having gone there shortly after his graduation in 1852 from what was then the Buffalo Medical College. He was born in Akron, N. Y., and received his grammer and high school education in that village. In 1852 he was appointed resident physician at the Sisters' Hospital, after which service he moved to Hamburg. In 1854 he was appointed surgeon of the 57th regiment of the National Guard. When the civil war broke out he was made colonel of the 98th regiment of volunteers and as commander of that organisation served throughout the war. In 1856 he became a member of the Erie County Medical Society and served as vice-president and president from 1864 to 1866. He married in 1857, Julia Clark Church, who survives him. His living children are George Burwell, of Hamburg and Mrs. Peter Thompson. The funeral services were conducted by the Hamburg Masonic lodge.

